# A complex systems approach to the study of change in psychotherapy

**DOI:** 10.1186/s12916-020-01662-2

**Published:** 2020-07-14

**Authors:** Adele M. Hayes, Leigh A. Andrews

**Affiliations:** Department of Psychological and Brain Sciences, University of Delaware, 108 Wolf Hall, Newark, DE 19716 USA

**Keywords:** Complex systems theory, Dynamical systems, Network theory, Process-based psychotherapy, Psychotherapy research

## Abstract

**Background:**

A growing body of research highlights the limitations of traditional methods for studying the process of change in psychotherapy. The science of complex systems offers a useful paradigm for studying patterns of psychopathology and the development of more functional patterns in psychotherapy. Some basic principles of change are presented from subdisciplines of complexity science that are particularly relevant to psychotherapy: dynamical systems theory, synergetics, and network theory. Two early warning signs of system transition that have been identified across sciences (critical fluctuations and critical slowing) are also described. The network destabilization and transition (NDT) model of therapeutic change is presented as a conceptual framework to import these principles to psychotherapy research and to suggest future research directions.

**Discussion:**

A complex systems approach has a number of implications for psychotherapy research. We describe important design considerations, targets for research, and analytic tools that can be used to conduct this type of research.

**Conclusions:**

A complex systems approach to psychotherapy research is both viable and necessary to more fully capture the dynamics of human change processes. Research to date suggests that the process of change in psychotherapy can be nonlinear and that periods of increased variability and critical slowing might be early warning signals of transition in psychotherapy, as they are in other systems in nature. Psychotherapy research has been limited by small samples and infrequent assessment, but ambulatory and electronic methods now allow researchers to more fully realize the potential of concepts and methods from complexity science.

## Background

There are now numerous forms of psychotherapy with demonstrated efficacy, but less is known about how these treatments have their effects—the process by which change occurs [1–3]. An understanding of factors that facilitate and inhibit therapeutic change can guide efforts to improve treatment efficacy and address the significant problems of relapse and recurrence. The science of complex adaptive systems offers theories and methods for studying change across a wide variety of physical and natural systems, ranging from cells and neurons, political and economic systems, weather patterns, and entire ecosystems. This science of system change also has great potential to inform the study of how psychotherapy has its effects, how data are collected and analyzed, and how to conceptualize and intervene in treatment.

There have been a number of early attempts to translate concepts of complex systems science into psychology and psychiatry [4–10], but uptake into the mainstream has been slow. One barrier has been the traditional randomized controlled trial (RCT) design, which is valuable for evaluating treatment efficacy, but the treatments have tended to emphasize single components of functioning (e.g., cognitions, emotions, behaviors, physiology) rather than the multi-component patterns of interest in complex systems research [3]. Until recently, symptom change was often analyzed in snapshots of symptoms at pre- and post-treatment with the assumption of gradual and linear change. It has become apparent that this traditional approach does not fully capture the patterns of psychopathology and the dynamics of therapeutic change [11–16]. Sciences that study complex systems have shifted from reductionist analyses of component parts and simple linear change to the study of interconnected elements and feedback loops that form patterns that evolve over time, often in nonlinear ways.

Another barrier to the application of a complex systems approach is that the subdisciplines relevant to psychotherapy research, including dynamical systems theory, self-organization and synergetics, and network theory, use somewhat different concepts and jargon, making it difficult to detect common themes and principles from the research. In addition, some of the early efforts [5, 7, 8] to import concepts and methods from complex systems sciences were ahead of the times. Psychotherapy researchers collaborated with physicists and presented detailed mathematical formulae, sophisticated time-series analyses, and computational modeling, which are standard in physics but were uncommon in psychotherapy research at the time. In addition, many of the studies presented cases with thousands of data points, which seemed unattainable in psychotherapy research and starkly different from the RCT design. Technological and methodological advances (e.g., electronic and web-based data collection, mobile and wearable devices, text mining programs) are now available to collect high density data, and a complex systems approach to psychotherapy research is gaining momentum.

The purpose of this article is to further advance a complex systems approach to psychotherapy research by presenting some basic principles in a way that is true to complexity science, yet also accessible to a wide range of researchers and clinicians. We present an integrative framework to help translate the concepts into a common language and to provide a structure for conceptualizing and studying different treatments and clinical problems, and perhaps for connecting psychotherapy research with other sciences.

### General principles of change in complex adaptive systems

Because we focus on common principles, it is not possible to capture all of the nuances specific to each subdiscipline. Rather, we distill some generic principles and common themes to facilitate the transfer and uptake of complex systems ideas to psychotherapy research.

#### Pattern formation and attractors

As we have presented elsewhere in the context of depression [17, 18], a dynamic system is a set of interconnected elements that evolve over time and self-organize into higher-order functional units [19], called *attractor states*, that are preferred and govern system behavior. Self-organization is the process by which lower-order processes interact and higher-order patterns emerge and then influence the lower-order processes in a top-down manner [13, 20–23]. Attractor states constrain system behavior such that it tends to be “pulled” back to these states when perturbed. An adaptive system is flexible as conditions change, but also able to maintain functional integrity in the face of perturbation [24]. A system that has multiple functional patterns (known as multistability) can flexibly switch between patterns to meet the demands of internal and external challenges [25].

Attractors that are well-established have strongly interconnected elements, with reinforcing and inhibiting feedback loops that can increase or decrease the probability of activation over time and contexts [18, 19, 21–23]. When attractor patterns are entrenched, they become rigid and relatively insensitive to challenges or new information. Significant disturbance or strong jolts are therefore required to disrupt these patterns. Less developed or destabilized attractors have a weaker hold, allowing the system to more easily switch to alternative states [25, 26].

#### System change: tipping points and nonlinear transitions

To maintain coherence, complex systems can adapt to, incorporate, or defend against challenges. Both deterministic (purposeful or causal) and stochastic (naturally occurring random events, fluctuations, noise) forces influence the maintenance and disruption of complex systems [15]. The probability of transition from one attractor to another depends on the strength of that attractor, the type of perturbation, the parameters that control system organization (control parameters), and the strength of alternate attractors [19, 23, 25, 26].

Change can be incremental and gradual, with minor fluctuations and adjustments within the dominant pattern of functioning without shifting to another state or reorganizing. However, when the control parameters that influence the movement of the system reach a critical threshold or “tipping point,” the dominant state can shift suddenly [26, 27]. This type of change, which characterizes much of nature, is often abrupt and discontinuous, with periods of turbulence as attractors destabilize and create the potential for *phase or order transitions.* During these transitions, systems can reorganize into qualitatively new patterns of functioning, such as from a healthy state to a diseased state [28].

Knowing when a transition will occur can have important, and sometimes critical, implications. With warning of impending transitions, prevention or intervention strategies can be mobilized to facilitate or forestall the transition. For instance, early warning signs of symptom exacerbation or transition to disease can inform medical treatment decisions and can be a matter of life and death, as with sudden cardiac events or seizures [28]. For this reason, scientists have identified generic early warning signs that occur consistently and herald transition across a wide range of systems [29, 30]. Two such warning signs are *critical fluctuations* [15, 27, 31] (rising variability in system behavior that indicates that the system dynamics are breaking down) and *critical slowing* [26, 27, 29] (a slower return to baseline or recovery after perturbation). Transition can involve movement from healthy to maladaptive states, such as the rapid cascade from relative health to frailty that can occur in the elderly after a fall [26]. Transition can also move in the opposite direction. For instance, critical slowing has also been demonstrated to precede recovery with interventions, such as when overexploited marine systems are restored after conservation efforts [32].

More functional patterns, whether newly developed or available but latent, are relatively weak unless strengthened and stabilized with repeated activation across contexts and by amplifying feedback loops [23, 25, 33]. There can be a period of vacillation or “flickering [34, 35]” between attractors, until one state is strong enough to capture the system. As the new attractor strengthens, it can compete with or inhibit the pre-existing attractor to prevent a return to that state, or it can become the preferred or default state [27, 36, 37]. For instance, as one is trying to establish an exercise habit, the previous unhealthy habits of a harried but sedentary lifestyle are strong, whereas regular exercise is new and not yet established. The old patterns are easily activated and can pull the person into the rut of old habits. It is not until the new learning consolidates and is maintained in memory that this pattern becomes the new norm [25, 33].

Components and processes in complex adaptive systems can operate on different timescales. Some variables move slowly (e.g., over months or years, such as personality or developmental processes) and others more quickly (e.g., in milliseconds, seconds, hours, or days). Researchers can also focus on different levels of analysis from microanalytic (e.g., cells and neurons) to macroanalytic scales (e.g., communities, countries, and geographical regions) [12, 24, 26, 38].

### Application to psychotherapy: network destabilization and transition model

We developed the network destabilization and transition model (NDT) as an organizing framework to import concepts and principles from complexity sciences (dynamical systems theory, synergetics, and network science) to psychotherapy research. We also integrate into this framework principles of change from modern learning theory that are consistent with, but have not yet been connected with, principles of change in complex systems science. The NDT model was originally presented specifically in the context of treating depression [17, 18], but here, we broaden the perspective to apply to psychopathology in general and to consider different forms of therapeutic change. The intent is to stimulate new research ideas and provide a framework for understanding and organizing disparate sets of existing and forthcoming research findings related to complex systems ideas in psychotherapy.

**Fig. 1:**
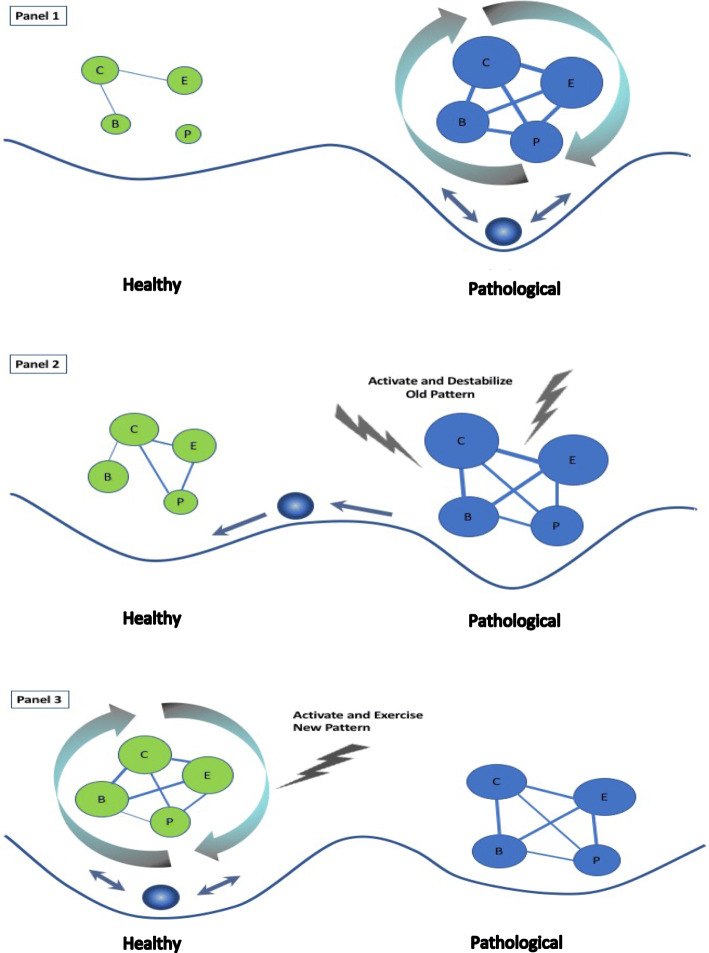
Network destabilization and transition (NDT) model. Attractor landscapes are depicted with the solid line and ball, which represents the state of the system. Pathological and more healthy networks are depicted with nodes of cognition (C), emotion (E), behavior (B), and physiological responses (P) and associated feedback loops. Larger nodes (circles) are stronger, and thicker lines represent stronger connections. **Panel 1** depicts a well-established pathological attractor with a hypothetical network that is strongly interconnected and maintained by amplifying feedback loops. **Panel 2** depicts a pathological attractor that is less strong. The adaptive attractor in **panel 2** is more developed than in **panel 1** and provides an alternative for the ball to enter when the pathological network is activated and destabilized. With repeated activation, exercise, and amplifying feedback loops (**panel 3**), the healthy attractor becomes stronger than the pathological attractor

Psychotherapy is in essence designed to promote new learning [1] to move a person from entrenched patterns of psychopathology to more flexible and functional patterns of functioning. Researchers have begun to conceptualize psychopathology as an attractor state with interacting elements of cognitions, emotions, behavior, and physiology [10, 14, 17, 39–43]. From this perspective, therapeutic targets are the patterns and processes that maintain clinical problems, rather than symptoms or isolated components of a larger system [3]. Tschacher and Haken [44] also highlight the importance of contextual factors, the therapeutic relationship, and environmental and random factors (stochastic variables), which also influence the change process. Therapist interventions (deterministic factors) can stabilize, shift, or destabilize attractors and also increase or decrease exposure to stochastic variables and their influence. As with change in other systems in nature, there are several routes to therapeutic change, which we illustrate with the NDT model in Fig. 1.

Principles of complex dynamic systems theory are often described in other sciences with metaphorical attractor landscape diagrams. With high-resolution time-series data, attractors, synchronization of elements, and critical fluctuations can be quantified and examined mathematically [44, 45]. Two possible attractor states of various strengths are depicted in panels 1–3 of Fig. 1, a pathological attractor (right side) and a healthier, more functional state (left side). Because attractors consist of multiple interacting elements, concepts from network science are useful to depict and study patterns that contribute to different attractors. We describe hypothetical pathological and healthy networks of cognitions, emotions, behaviors, and physiological functioning that have different numbers and strengths of connections, and also amplifying feedback loops that influence network strength.

It is important to note that more than two patterns can be relevant (e.g., multistability [25]) to psychopathology, and it is not clear whether pathological and more healthy states are best conceptualized as separate networks or as part of a single larger network. For illustrative purposes, we depict two hypothetical attractors and their associated networks. A two-attractor schematic is simple and can be clinically meaningful [46]. For instance, Haken and Tschacher [23] demonstrate in computer simulations that it is more potent to weaken a maladaptive attractor and develop and train just one alternative attractor than to diffuse efforts and create multiple weak attractors.

The right side of the first panel of Fig. 1 depicts a strong and entrenched state of psychopathology that forms a “rut” that is easy to enter and difficult to exit [46, 47]. The ball, which represents the state of the system, is caught in the pathological attractor. The thought-emotion-action-physiological patterns that make up the maladaptive attractor can be dense and strongly interconnected, especially with more recurrent and chronic disorders such as depression, bipolar disorder, and substance use disorders. Maintaining processes (e.g., rumination, avoidance, and other maladaptive emotion regulation strategies) can interfere with the processing of new information, creating feedback loops that perpetuate the pathological patterns and symptoms and make them difficult to destabilize [17, 48, 49]. In contrast, the components of more healthy functioning (left side) are sparse, weakly connected, and not likely to sustain if activated. It is difficult to capture the ball in the shallow well of what is only a potential attractor.

Change in psychotherapy can take a number of forms. One type of change involves making *minor adjustments* to maladaptive patterns [13, 44]. For instance, harm reduction strategies reduce some negative consequences of pathological patterns, but they do not induce fundamental attractor changes. An example of this kind of within-attractor change is to provide clean needles to intravenous drug users, which improves safety but does not address the addiction directly. Distress tolerance, mindfulness [50], and positive emotion activation approaches [51–53] can change the threshold of activation (or the sensitivity) and automaticity of both pathological and more functional patterns. Behavioral, interpersonal, cognitive reappraisal, emotion regulation, or parenting skills can be used to: (1) reduce feedback loops that block new information and interfere with new learning (spiral in panel 1, right), (2) deactivate or unhook from the pathological patterns of the attractor, or (3) compensate for or override these patterns (see also [1]). In addition, these skills can be used to decrease exposure to stochastic factors or reduce their influence [44]. All of these strategies work within the pathological attractor but do not change it directly.

Another type of change involves *switching* from a pathological to a more healthy attractor. To increase the probability of switching out of the pathological attractor, there must be an alternative to switch to. The healthy attractor on the left side of panel 2 has developed and is stronger than it was in panel 1. The therapist can provide a supportive environment and therapeutic alliance to increase the patient’s readiness, resources, and skills to develop (or reactivate) more healthy modes of functioning [54, 55]. An example of this approach is Beck’s recovery-oriented cognitive therapy for schizophrenia [56]. This intervention teaches patients to switch from a “patient mode” that is disorder-focused to an “adaptive mode” that focuses on positive beliefs, aspirations, strengths, and values of the individual. The adaptive mode is repeatedly activated and exercised to increase its accessibility and strength. Similarly, positive emotion activation approaches [51–53] can help build and strengthen a more healthy attractor. This alternative attractor increases the probability of movement from the pathological state to a healthier state, but again, the interventions do not change the pathological attractor directly.

Another type of change involves *destabilizing* the pathological attractor and developing new, more healthy patterns of functioning. If the pathological attractor is entrenched, interventions (and also stochastic factors) can activate and challenge it to provide the destabilizing jolt (panel 2, right side) needed to break the stasis and facilitate transition [23]. Exposure therapy, insight-oriented therapy, and emotion-focused and cognitive restructuring techniques are examples of interventions that introduce corrective information and skills to induce dissonance and perturb maladaptive networks [17, 57]. During this period of destabilization, indicators of critical fluctuations (increased variance) are likely to be apparent. This period can be characterized by system-wide disturbance and symptom exacerbation, but this turbulence can also increase the flexibility needed for attractor change [6, 11, 14]. Critical slowing might be apparent, as the person does not return as quickly to the pathological patterns after perturbation.

In panel 3, the pathological and more healthy attractors are now both options, and the person can move between them. This illustrates a case of bistability [15, 25, 58], when processes such as flickering can occur [34, 35]. A number of techniques are available to *stabilize new learning.* Therapists can create circumstances and introduce interventions that push or nudge the patient to the new, more healthy attractor; change its salience and threshold of activation; exercise it; and create upward spirals (panel 3, left side). These techniques can strengthen and generalize the new attractor, as well as make it easier and more automatic for the patient to respond differently in the future [58–60].

However, the weakened pathological network on the right side of the figure can still be activated by stressful life events and random stochastic forces, and it can have a very strong pull. The new attractor, if strengthened, can compete with or inhibit the old pattern, consistent with complex systems principles [25] and theories of change in psychotherapy related to inhibitory learning [61] and competitive retrieval [1, 62]. Techniques are available to enhance inhibitory learning (e.g., repetition, practice in different contexts and variable conditions [61]) and to improve memory flexibility and retrieval of the new learning [62–64]. With repeated activation (panel 3, left side) across contexts, the new attractor can become stronger and more interconnected, whereas the pathological pattern can weaken and its elements become more decoupled. Feedback loops can amplify and reinforce new patterns (spirals, left side), similar to the idea of creating upward spirals of continued growth [59, 60, 65].

## Discussion

A complex systems approach has a number of important implications for psychotherapy research, including the need for intensive longitudinal data, the study of discontinuous and nonlinear change, and a focus on patterns of functioning rather than single components. We describe each of these topics in turn and provide examples of how this research can be conducted, avenues for future research, and some useful analytic tools that can be used.

### Data collection considerations

#### Timescale

An important design consideration when collecting time-series data is selecting the time interval (sampling rate) most sensitive for detecting change in the variables of interest [5, 66]. Some variables change slowly (e.g., over weeks, months, years) and others more quickly (e.g., in seconds, minutes, hours) [24, 31, 67–70]. Researchers must select the sampling rate that considers theory and what is known about how a given variable moves across time. For instance, perspective shifts and meaning making might unfold over several sessions of therapy, and therefore, a weekly interval might be most appropriate, whereas emotions might move frequently at daily or hourly intervals. Methods are now available for analyzing variables on different timescales. Duncan et al. [68] demonstrate how dynamical systems simulations can use differential equations to model oscillations between states of craving and depressed mood (rapidly changing states) with recovery (slow process) and relapse (a rapid and abrupt event). Tschacher and Haken [15] also provide detailed equations and computer simulations to demonstrate concretely how to model therapy variables at different timescales.

#### Breadth and duration of assessment

Microanalytic assessments, such as those conducted on a timescale of minutes or multiple times per day, typically include only a few items assessed over 1- to 2-week periods. The sampling rate is high, but the tradeoff is that the number of variables and duration of assessments are low. Ideally, measurement of pathological patterns and symptoms would occur over the course of therapy, between sessions, and after therapy to capture therapeutic change as it unfolds and generalizes. Assessment in the period after therapy has been particularly sparse with tradition clinical trial designs.

There are ways to increase data density by including less burdensome methods, such as gathering passive data (e.g., activity level, exercise, sleep, and social media usage with wearables and smartphones), written text or speech to analyze with text mining or other content analysis methods [71–73], and bursts of assessment triggered at particularly important times (e.g., during periods of high risk). Helmich and colleagues [74] designed a study that exemplifies the use of multiple measures collected at different sampling rates and periods of time. Mood was assessed five times per day for 4 months using smartphone experience sampling, heart rate and actigraphy data were gathered in time bursts using wearable devices, and depression symptoms were assessed weekly using self-report measures. They describe how to combine these different data streams to examine whether early warning signs (autocorrelation, variance, and overall network connectivity) increase before large transitions in depression symptoms.

#### Level of analysis

Complex systems research is in essence an idiographic or individual-level approach to the study of system change. Much of psychotherapy research has been conducted at the nomothetic level of group averages, but it has become increasingly apparent that findings from one level might not generalize to the other [70, 75]. Individual-level data allow for an examination of the dynamics of a given person, which can have direct clinical relevance, but an important task of science is also to detect patterns and principles that generalize across people and across studies. Identifying common indices of early warning signals across systems and sciences is an example of bridging both levels of analyses [26]. Ellison and colleagues [70] illustrate how to combine levels of analysis in one study, using ecological momentary assessment methods and Group Iterative Multiple Model Estimation (GIMME). They demonstrate how to identify dynamic patterns in time-series data at various levels (individual, subgroup, and group) in the context of borderline personality disorder.

### Discontinuous and nonlinear change

#### Different trajectories of change

A complex systems approach calls for the study of different trajectories of symptom change (or other outcome variables). A typical assumption in psychotherapy research is that change is gradual and linear, but individual time course data has revealed that the process of psychotherapy can also follow a nonlinear course [11]. Change in symptoms and therapy processes can show quadratic patterns (U- or V-shaped [76]), as well as cubic [77, 78], saw-toothed [79, 80], and other nonlinear patterns [81–83], all of which have been associated with better treatment outcomes. In addition, specific types of discontinuities or sudden jumps in time course data have been identified that predict symptom reduction across a variety of clinical disorders, including the sudden gain [74, 84–86] (a large decrease in symptoms in a 1-week interval), the depression spike [78] (an intentionally induced and transient symptom exacerbation), and the cusp catastrophe pattern, which has been used to model sudden transitions from an abstinent state to relapse in addiction research [87, 88]. Together, these studies challenge the assumption that sudden changes, or even periods of symptom worsening, indicate that the patient is “off track” and in need of course correction [66].

#### Early warning signals

A complex systems approach views periods of increased variability and turbulence in psychotherapy as potential opportunities for change [89] rather than as noise or disturbances to quell. An exciting endeavor is to explore whether human change processes operate in ways that are similar to those in other living systems. Critical fluctuations and critical slowing have been well-documented to herald transition across a number of systems in nature [20, 27, 29, 30], and some research suggests that early warning signs can be measured in various ways in psychotherapy and that these signals might matter [90].

Too few studies have been conducted on early warning signals in psychotherapy to draw firm conclusions, but the findings thus far suggest that nonlinear and discontinuous changes and periods of rising variability in symptoms and patterns of pathology occur in psychotherapy, and that these fluctuations predict symptom reduction and the development of more healthy patterns of functioning. For example, periods of increased fluctuation in symptoms or therapy process variables have been found to predict both sudden gains [91, 92] and sudden losses [92] in treatments for depression. In observational coding of early sessions of cognitive therapy (CT) for depression, more destabilization across components of a maladaptive depressive pattern predicted more improvement in depressive symptoms at post-treatment [4]. Similarly, more variability across cognitive, affective, and behavioral domains of functioning before sudden gains predicted better depression outcomes at 12-month follow-up in a sample with treatment-resistant depression [93]. Using a program called GridWare [94, 95] to quantify dispersion or movement of variables in a phase space (representation of possible states of a system), more variability in a pathological pattern with cognitive, affective, and behavioral components predicted improvement in personality disorder symptoms and also more healthy functioning after CT for personality disorders [96].

Recurrence quantification analysis [97] has been used to quantify change in the rigidity and repetition of communication patterns in children receiving cognitive behavioral therapy (CBT). Lichtwarck-Aschoff and van Rooij [98] reported that an increase in the variability of rigid and inhibited child-therapist communication patterns predicted not only symptom reduction in anxiety, but also more adaptive, prosocial communication. Similarly, more variability in maladaptive parent-child interactions predicted more symptom and behavioral improvement in aggressive children [99].

Another way to measure critical fluctuation is to calculate dynamic complexity [100], which considers the strength, number, and distribution of fluctuations in time-series data [31]. Critical fluctuations (higher dynamic complexity) in daily self-ratings of psychotherapy process variables have been shown to precede and predict symptom reduction in patients with obsessive-compulsive disorder [54, 101] and mood disorders [102, 103]. A program for calculating dynamic complexity is available in R statistical package [100]. The Synergetic Navigation System (SNS) is an example of a data collection system that can be used to collect multiple variables over the course of treatment and after [45]. The SNS not only calculates recurrence plots of the clinical patterns of interest and critical fluctuations (dynamic complexity) of symptoms and other variables, but it also visually depicts these variables in graphs that can be used to provide ongoing feedback to patients across the course of treatment.

A system that is on the verge of transition will also show critical slowing, where it requires more time to recover from small perturbations [26, 30]. Very little research has been conducted on critical slowing in the context of psychotherapy. Wichers et al. [104] investigated critical slowing in an intensive longitudinal case study of a patient with a history of depression, who experienced a depressive episode after discontinuing his medication. His mood ratings showed indicators of critical slowing (increased temporal autocorrelations, increased variance, and stronger intercorrelations) before the onset of the depressive episode. This pattern of findings held consistent in subsequent re-analyses of the same data with more sophisticated tests of correlational change in longitudinal data [105, 106]. Early warning sign toolboxes are available for detecting signals of critical slowing in R statistical package [107, 108], and a number of studies are currently underway.

A particularly interesting task will be to examine the extent to which early warning signals predict transition across a range of clinical problems and treatments and might represent a principle of therapeutic change. Future research can continue clarify how to distinguish fluctuations that are noise, random variation, or personality-based lability (e.g., neuroticism, affective lability) from those that predict system transition [74, 81]. It is not clear whether the signatures of early warning signals differ when predicting the onset of clinical disorders, improvement or worsening in psychotherapy, or relapse. It is clinically important to differentiate turbulence that precedes the degradation or worsening of a system from turbulence that heralds change to a more functional state. Early warning signals seem most relevant to the type of change that involves destabilizing one attractor and moving to another (Fig. 1, panel 2). It is not yet known whether these indicators also apply to other types of change described in the NDT model, such as switching from one attractor to another without changing the pathological attractor directly (e.g., switching from “patient mode” to “adaptive mode” in Beck’s recovery-oriented cognitive therapy for schizophrenia [56]).

Turning points in symptom trajectories and early warning signals can be used in psychotherapy research to isolate key change processes in a course of treatment [11]. For example, our research group has examined a patient variable called cognitive-emotional processing (considering different perspectives, making meaning, and shifting perspectives and affective responses) at three points of discontinuity (immediately before sudden gains in depression scores [93, 109], after a depression spike [78], and during a period of increased variability in maladaptive personality patterns [96]). More processing at each of these change points predicted better treatment outcomes in the three studies, consistent with the view that processing might be a potential mechanism of change across treatments [57].

Personal early warning signs can be used to give patients ongoing feedback on vulnerability and resilience, especially when the risk of relapse is high [45]. Techniques such as “just-in-time” adaptive interventions can be tailored and deployed for a specific person, based on that person’s early warning signals, as is being done following treatment for self-harm, substance abuse, and schizophrenia [110] and for risk of suicide [111]. Early warning signs can also be used to activate intensive assessment of variables that might be associated with clinically significant transitions, such as moving from the urge to self-harm to cutting oneself. Research with this kind of precise time lock can help inform prevention efforts.

### Patterns and feedback loops

Another implication of complexity science is that attractors consist of multiple interconnected elements, so patterns are the variables of interest rather than single or a few separate components. As described earlier, recurrence quantification analysis [97] and related analytic tools such as GridWare [94, 95] and the Synergetic Navigation System [45] can be used to capture multi-component patterns for individuals (e.g., entrenched pathological patterns related to personality disorders [96]) or dyads (e.g., rigid and inhibited child-therapist communication patterns [98]) over specified windows of time, as well as new patterns learned in treatment (e.g., more healthy patterns of functioning [96], more prosocial communication patterns [98]).

Burger and colleagues [112] present a sophisticated computational model for a hypothetical patient with panic disorder that illustrates how to use nonlinear differential equations to examine *functional* relationships between and change in cognitive, emotional, behavioral, and physiological components of a pathological pattern that maintains panic symptoms. Reinforcing and inhibiting processes are also included, similar to the feedback loops presented in the NDT model (Fig. 1, panels 1 and 3). In addition, the effects of two interventions (exposure and cognitive reappraisal and the combination) are examined in simulations. The model also includes new learning, such as more functional interpretations of stimuli, which compete with and lessen the impact of catastrophic interpretations on panic symptoms. This approach could be useful for future research because it allows for the analysis of multi-component patterns, feedback loops, interventions, and new learning in the system, all components of complex systems research.

The analytic methods from dynamical systems and synergetics research assess the strength and repetition of patterns, but they provide less information on the architecture and connectivity of the components. Network analyses to date have focused primarily on cross-sections of data and symptom-based networks that do not capture mechanisms and processes that might generate symptoms [3]. However, it is possible to include networks of cognitive, emotional, behavioral, and physiological components that contribute to psychopathology, as well as new learning and patterns that develop in treatment. Network analysis tools can quantify the structure, density, connectivity, and threshold of activation of patterns and how they change over time [113–115]. Network analysis provides tools to depict and measure patterns of psychopathology for a given sample and also personalized for a specific individual [10, 40, 43, 113–116], with the potential to guide treatment decisions and selection [112, 117–119].

A limitation of a number of network analysis tools is that they assume stationarity—that each variable over time demonstrates a similar mean, variance, and relationship with other variables and with itself [115, 120]. The very nature of psychotherapy, however, is to induce change. Methodologists have developed ways to examine networks in windows of relative stability, such as the beginning and end of treatment [121], and to include intervention as a variable in network models to examine treatment effects on symptoms at different time windows [122]. More recently, analytic strategies are being developed to examine nonlinear jumps and regime (attractor) changes using network analyses [105, 123]. Tschacher and Haken [15] describe methods from dynamical systems science and synergetics to examine effects over time of one component in a system on another component, using nonlinear differential equations. They also describe how to conduct simultaneous and cross-lagged correlations between variables in specified windows of time (e.g., a week) in time-series data, as well as correlations between window-wise slopes of different variables.

As these tools are refined to model network change and nonlinear dynamics, the kinds of questions that psychotherapy researchers can examine expand considerably. As proposed in the NDT model, therapeutic change can involve making within-attractor modifications, switching from one existing attractor to another, and destabilizing the old and developing a new more functional pattern. Research that includes both pathological and more functional patterns can examine the type of change different therapies tend to induce, from incremental shifts to the development of new, more healthy patterns [1]. This can be examined at both the individual and aggregate levels. Another question is whether the density and connectivity of components in the pathological and more healthy patterns are altered with treatment, as depicted in the NDT model for the destabilizing type of change (Fig. 1, panels 2 and 3). Related to this, the NDT model depicts two attractors with associated pathological and positive networks, but it is possible that a more accurate representation is a single network with excitatory and inhibitory pathways, as in the Burger et al. model [112]. More research that considers networks of both old and new learning can help to clarify this.

### Interplay of pathological and new patterns of learning

Another aspect of complex systems is that as new attractors develop, they can be strengthened and compete with or inhibit old attractors (Fig. 1, panel 3). Although they do not reference complex systems theory or competition between attractors, modern cognitive and learning theories similarly suggest that psychotherapy promotes new learning [1] and that instead of replacing pathological patterns, new patterns can compete with or inhibit the old to reduce the risk of relapse [61, 62, 124]. Very little research has directly examined this old-new attractor competition principle in the context of psychotherapy, even though it has important implications for stabilizing change during treatment and for preventing relapse.

Two studies based on observational coding of therapy sessions found that more new learning expressed in CBT sessions did not directly predict treatment outcomes, but instead interacted with pathological processes to lessen their negative impact. In a study of cognitive therapy for treatment-resistant depression, more rumination and avoidance after a spike in depression scores predicted worse 12-month depression outcomes, but not for those who also showed new learning and flexibility across cognitive, emotional, and behavioral domains of functioning [93]. A similar interaction of old and new was reported in a sample of traumatized youth who received a trauma-focused treatment. More overgeneralized negative beliefs during the trauma processing phase of treatment predicted worse 12-month outcomes on externalizing symptoms, but not for those who also developed new, more healthy beliefs (accommodation) [125]. Accommodation alone did not predict outcomes, so its influence seemed to lessen the impact of overgeneralization. These statistical interactions are only rough approximations for studying how new learning might compete with or inhibit pathological processes, but these concepts could be tested further using more sophisticated methods.

Garland et al. [126] describe how upward spirals of positive emotion can be used to counter downward spirals of negativity, and perhaps similar methods could be applied to psychotherapy research. Hoorelbeke et al. [120] used network analysis to examine how both risk and protective factors were associated in a sample with remitted depression, and this approach might be used to study interactions of pathological and healthy patterns over the course of therapy and after. Burger et al.’s [112] computational model of an individualized pathological pattern that maintains panic symptoms demonstrates how functional beliefs learned in therapy can be modeled as inhibitory factors that compete with the credibility of the catastrophic beliefs and thus reduce their impact. Haken and Tschacher [23] also provide a useful computational model to illustrate concretely how to model two attractors that compete, and how therapy can strengthen the new attractor to have a higher affective and motivational valence than the old attractor so that it becomes dominant. These tools could be particularly helpful for future research on the interplay of old and new learning in psychotherapy, which could provide more direct tests of inhibitory learning [61, 124] and competitive retrieval [62] theories.

## Conclusion

The NDT model of therapeutic change [17, 18] is presented as a conceptual framework to distill and translate concepts from subdisciplines of complexity science (dynamical systems theory, synergetics, and network theory) and to integrate them with related principles of therapeutic change from psychotherapy research. We use a common language and structure to facilitate the transfer. We describe examples of studies that have begun to examine principles of change from a complex systems perspective, as well as data collection and analytic techniques that can advance this type of research. The NDT model might provide a common structure for studying and organizing findings related to topics of complex systems science, such as the types of perturbation that can induce different types of change (incremental to qualitative transition), early warning signs of transition, how and whether network change and transition occur across a range of treatments and disorders, and how to promote and sustain adaptation and well-being (or health) after treatment. These concepts and methods can add to the traditional clinical trial design to reveal the dynamics of therapeutic change, in addition to evaluating the efficacy of treatments. A common organizational structure might also facilitate comparisons of findings from psychotherapy research with those in other sciences, as Scheffer and colleagues [26, 29, 30] did when they identified generic early warning signs common across different systems and methods. Nearly 30 years after complex systems approaches were introduced to psychotherapy researchers, the time is now ripe to bring them to the mainstream.

## Data Availability

Not applicable

## Abbreviations

CT: Cognitive therapy
CBT: Cognitive behavioral therapy
NDT: Network destabilization and transition
RCT: Randomized controlled trial
SNS: Synergetic Navigation System
